# Retracted: Contamination of Hospital Water Supplies in Gilan, Iran, with *Legionella pneumophila, Escherichia coli,* and *Pseudomonas aeruginosa*

**DOI:** 10.1155/2016/4801509

**Published:** 2016-10-12

**Authors:** Interdisciplinary Perspectives on Infectious Diseases

Interdisciplinary Perspectives on Infectious Diseases has retracted the article titled “Contamination of Hospital Water Supplies in Gilan, Iran, with* Legionella pneumophila*,* Escherichia coli*, and* Pseudomonas aeruginosa*” [1]. The article was found to contain a substantial amount of material, without citation, from previously published articles.
